# Poly(ADP-Ribose) Glycohydrolase (PARG) Silencing Suppresses Benzo(a)pyrene Induced Cell Transformation

**DOI:** 10.1371/journal.pone.0151172

**Published:** 2016-03-22

**Authors:** Xuan Li, Xiyi Li, Zhiliang Zhu, Peiwu Huang, Zhixiong Zhuang, Jianjun Liu, Wei Gao, Yinpin Liu, Haiyan Huang

**Affiliations:** 1 Key Laboratory of Modern Toxicology of Shenzhen, Shenzhen Center for Disease Control and Prevention, Guangdong, China; 2 School of Public Health, Guangxi Medical University, Guangxi, China; 3 Department of Occupational Disease Prevention, Baoan Center for Disease Control and Prevention, Guangdong, China; University of South Alabama Mitchell Cancer Institute, UNITED STATES

## Abstract

Benzo(a)pyrene (BaP) is a ubiquitously distributed environmental pollutant and known carcinogen, which can induce malignant transformation in rodent and human cells. Poly(ADP-ribose) glycohydrolase (PARG), the primary enzyme that catalyzes the degradation of poly(ADP-ribose) (PAR), has been known to play an important role in regulating DNA damage repair and maintaining genomic stability. Although PARG has been shown to be a downstream effector of BaP, the role of PARG in BaP induced carcinogenesis remains unclear. In this study, we used the PARG-deficient human bronchial epithelial cell line (shPARG) as a model to examine how PARG contributed to the carcinogenesis induced by chronic BaP exposure under various concentrations (0, 10, 20 and 40 μM). Our results showed that PARG silencing dramatically reduced DNA damages, chromosome abnormalities, and micronuclei formations in the PARG-deficient human bronchial epithelial cells compared to the control cells (16HBE cells). Meanwhile, the wound healing assay showed that PARG silencing significantly inhibited BaP-induced cell migration. Furthermore, silencing of PARG significantly reduced the volume and weight of tumors in Balb/c nude mice injected with BaP induced transformed human bronchial epithelial cells. This was the first study that reported evidences to support an oncogenic role of PARG in BaP induced carcinogenesis, which provided a new perspective for our understanding in BaP exposure induced cancer.

## Introduction

The chemotherapeutic potential in targeting the metabolism of poly(ADP-ribose) (PAR) biopolymers in cancer cells has been proposed because of the fundamental role of PAR in maintaining genomic integrity [1]. PAR is synthesized primarily by poly(ADP-ribose) polymerase-1 (PARP-1) and PARP-2 [2,3]. Once synthesized, PAR is mainly catabolized by the poly(ADP-ribose) glycohydrolase (PARG) through hydrolysis [4,5]. The coordinated action of PARPs and PARG is required for proper cellular responses to DNA damages and maintenance of genomic stability [6–8].

PARG has been associated with various cellular processes, including the cellular response to oxidative stress and apoptosis [9,10]. The PARG-null mutation has been linked to increased levels of DNA damage, cell death, genomic instability, and chemosensitization to sublethal doses of DNA-damaging agents [11–13]. PARG-deficient mouse embryonic fibroblasts (MEFs) and PARG full length isoform deleted mice show increased sensitivity to alkylating agents and *γ*-irradiation [14]. In addition, PARG deficiency sensitizes tumor cells to chemo- and radiation therapies [15,16]. Silencing PARG has also been shown to inhibit the growth of human colon cancer cells *in vitro* [17] and reduce the number of liver metastases in a murine model of colon carcinoma [18]. Previous studies have reported that Inhibition of PARG can lead to cell death in BRCA2-deficient tumor cells [19]. These studies provide promising evidences to support that PARG is a potential interventional target to improve the efficacy of cancer chemotherapy. However, the underlying molecular mechanism in PARG mediated cancer development and progression is still elusive, which prohibits the possible clinical applications of PARG in cancer therapy.

Benzo(a)pyrene (BaP), one of the most widely studied polycyclic aromatic hydrocarbons (PAHs), is a known carcinogen and can cause DNA damage, chromosome abnormalities, and cell death [20]. Our previous data had shown that BaP-induced cell death was mediated by PARG. Down-regulation of PARG protected cells from the cytotoxic effects of BaP, probably by regulating the ATM/p53 pathway and the metabolic activation of BaP [21]. In addition, PARG silencing inhibited BaP induced changes of DNA methyltransferase (DNMT) activity [22]. These findings indicated that PARG played a role in BaP induced carcinogenesis.

In our previous study, we found that suppression of PARG attenuated the DNA damages induced by BaP in a human bronchial epithelial cell line, in which the expression of PARG was stably silenced by lentivirus-mediated RNA interference.[21]. In this study, we aimed to determine the role of PARG in the carcinogenesis induced by BaP. We discovered that PARG played an important role in BaP induced malignant cell transformation. PARG silencing significantly reduced DNA damage, chromosome abnormalities, cell migration, and colony formation in 16HBE cells exposed to BaP. Our results provided novel evidences to support the oncogenic role of PARG in BaP mediated carcinogenesis.

## Materials and Methods

### Cell culture and BaP-induced cell transformation

The human bronchial epithelial cell (16HBE cell) was a gift from Dr. Weidong Ji (Sun Yat-Sen University, Guangzhou, China) [23]. The PARG-deficient human bronchial epithelial cell (shPARG cell) was generated from 16HBE cell stably expressed PARG shRNA in our previous study [21]. Cells were cultured in MEM containing 10% fetal bovine serum (FBS) and 100 units/ml penicillin/streptomycin at 37°C in a humidified atmosphere with 5% CO_2_. According to our previous study [21], cells grown to 80% confluency were treated with 0, 10, 20, or 40 μmol/L BaP for 24 hrs once a week for indicated length of time.

### Western blot analysis

Total proteins were extracted from cells in different treatment groups and the concentrations were measured by the BCA kit. Protein samples (30 μg/lane) were separated by SDS-PAGE and transferred to PVDF membranes. The membrane was then incubated overnight at 4°C with different primary antibodies in blocking solution: mouse monoclonal anti-PADPR 10H (abcam, Cambridge, UK); mouse monoclonal anti-PARG antibody (sc-398563, Santa Cruz); mouse monoclonal anti-GAPDH (sc-32233, Santa Cruz). The immunoblots were then incubated with goat anti-mouse IgG-HRP (sc-2005, Santa Cruz) as secondary antibody followed by ECL detection (GE Healthcare).

### Soft agar colony formation assay

The soft agar colony formation assay was performed in 6-well plates using a soft agar colony formation assay kit (GMS10024, GenMed) following the manufacturer's instructions. Briefly, the 16HBE and shPARG cells for different treatment groups (3,000 per group) were seeded in the soft agar gel and cultured at 37°C in a 5% CO_2_ incubator for 3 weeks. The colonies of different groups were scored using a microscope and Image-Pro PLUS 6.0 software.

### Comet assay

DNA damage was measured following the alkaline procedure described by Singh [24] with minor modifications. The single-cell suspension in PBS (1×10^6^/ml) were mixed with 4 times of 0.6% low melting point agarose and then immediately spread on microscope glass slides pre-coated with 0.7% normal melting point agarose. The mixture was allowed to form a gel and then soaked with alkaline lysis buffer (10 mMTris, 2.5 M NaCl and 100 mM Na_2_-EDTA plus 10% DMSO and 1% Triton X-100, pH 10.0) at 4°C for 2hrs. Next, the cells were treated with fresh and pre-cooled electrophoretic buffer (300 mM NaCl and 1 mM Na_2_-EDTA, pH 13.0) for 20 min to allow the DNA to unwind. Electrophoresis was then performed on ice at 25 V and 300 mA for 25 min. At the end of electrophoresis, the slides were neutralized with Tris-HCl buffer (0.4 M Tris, pH 7.5) and stained with 5 μg/mL propidium iodide. Stained comets were examined and photographed by a fluorescence microscope (IX51, OLYMPUS). All steps described previously were carried out in darkness. DNA comets were evaluated by measuring the percentage of tail DNA, tail length, tail moment (TM), and olive tail moment (OTM) of 100 comets using the Comet Assay Software Project (CASP). A higher TM indicated a higher level of DNA damage.

### Chromosome aberration (CA) assay

Cells of different groups (treated with different concentrations of BaP for 1, 9, and 15 weeks) were cultured in fresh media until they reach logarithmic growth phase. Colchicines (Merck) were added to the cell culture medium for metaphase arrest at a concentration of 0.1 μg/mL for 12 hrs. The cells were harvested by trypsin and resuspended in 75 mM KCl before being fixed in freshly prepared acetic acid-methanol (1:3). At least three changes were given in fixative before the cell suspension was dropped into a pre-cleaned chilled microscopic glass slide and dried at room temperature. Slides were then stained with Wright-Giemsa stain for 20 min. Well-spread cells in metaphase were photographed by an Olympus CX41 microscope at 100x magnification with a Meta Systems camera.

### Cytokinesis-block micronuclei (CBMN) assay

Cells of different groups were cultured in fresh media. Cyt-B (3.5 μg/mL, 48 hrs at 37°C) was added to halt cell division in the binucleate stage. The cells were then harvested by trypsinization, hypotonically treated (75 mM KCl, 20 min at 37°C), and fixed in freshly prepared acetic acid-methanol (1:3). The cell suspension was dropped to a pre-cleaned glass slides and dried at room temperature. Giemsa staining was used for scoring micronuclei under a bright-field microscope with 100-fold magnification (Olympus, Austria). For each sample group, experiments were performed for at least twice and two slides of each sample were examined. The frequency of micronuclei (MN) was calculated based on the criteria published by Fenech et al [25].

### Evaluation of cell migration by wound healing assay

Cells were plated in 6-well plates (1×10^6^ cells/well) and allowed to reach 80% confluency. Wounds were made by scratching the cell layer using a 100 μL sterile pipette tip along a ruler. Unattached cells and debris were removed thoroughly by washing the scratched area repeatedly with PBS. In the presence of serum, cells were cultured for 24 hrs after scratching. Images were acquired by a Nikon TS-100F inverted microscope (100× magnification). The images shown in this study are representative of three independent experiments. The images were analyzed by Image-Pro PLUS 6.0 software.

### Tumorigenicity assay in nude mice

Female Balb/c nude mice (aged 5 weeks) purchased from the Experimental Animal Center of Guangdong Province (Guangzhou, China). Mice were housed 5 per cage with free access to food and water and maintained at ambient temperature (22°C) with a 12:12 h light/dark cycle in the animal facility of Shenzhen Center for Disease Control and Prevention. All mice were randomly divided into four groups (n = 5 in each group).

Cells from different groups in exponential growth phase were harvested and resuspended in Ca^2+^- and Mg^2+^-free HBSS at 5×10^5^ cells/50 μL. Cell viability was determined by trypan blue exclusion, and only single-cell suspensions with >95% viability were used for subcutaneous injections. Female Balb/c mice were implanted with 2×10^5^ 16HBE, 40 μM BaP/15W-16HBE, shPARG, and 40 μM BaP/15W-shPARG cells in 20 μL of Ca^2+^- and Mg^2+^-free HBSS. Four weeks after the implantation, the mice were euthanized and necropsied. Body weight and tumor weight were recorded. Tumor volumes were measured with calipers and calculated following formula, V (mm^3^) = (H×W×L) ×0.52 (H = height, W = width, and L = length of the tumor). All animal experiments were approved by the Committee on the Ethics of Animal Experiments of Shenzhen University. All forms of surgery were performed under sodium pentobarbital anesthesia, and all efforts were made to minimize animal suffering.

### Pathological analysis of tumor tissue

Biopsied tumor tissues were dissected out from the mice and immersed immediately in isopentane cooled in liquid nitrogen. Serial cryostat sections were cut at a thickness of 10 μm and stained by hematoxylin-eosin (H&E). Images were acquired using an Olympus CX41microscope.

### Immunofluorescence analysis of PARG

For immunofluorescence analysis, tumors were dehydrated in 30% sucrose, embedded in optimum cutting temperature (OCT) compound, and divided into 15-μm-thick sections. After blocking with bovine serum albumin, the slides were incubated overnight with the mouse monoclonal anti-PARG antibody (sc-398563; Santa Cruz) at 1:100 dilutions. The samples were subjected to immunofluorescent staining with goat anti-mouse IgG-FITC (sc-2010; Santa Cruz) and DNA counter staining with DAPI. Images were acquired by a confocal laser scanning Microscope (Leica Tcs sp5, Leica Microsystems).

### Statistical analysis

The experiments were conducted at least three times in duplicate. Biostatistical analyses were performed using SPSS 17.0 software. Differences between groups were analyzed by the Student’s t-test and one-way ANOVA. Statistical significance was considered when *p*<0.05.

## Results

### PARG silencing down-regulated cell colony formation induced by BaP

**Fig 1:**
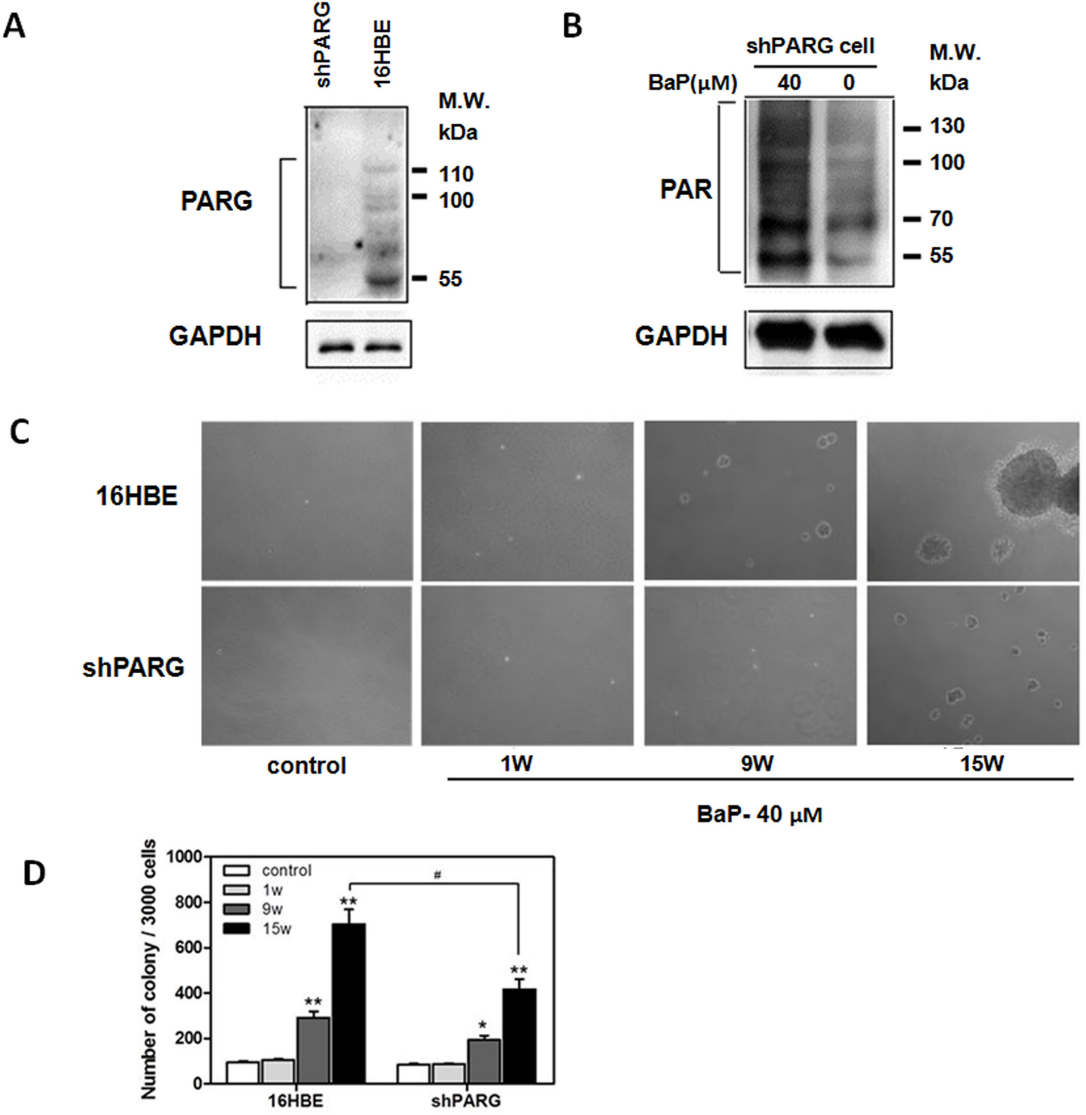
PARG silencing down-regulated cell colony growth induced by BaP exposure. **A**, Protein expression of PARG in shPARG and 16HBE cell which cultured at the 15th week was determined by immunoblotting. **B**, Protein expression of PAR in shPARG cell which cultured at the 15th week treated with 40 μM BaP for 24 hrs. Detection of total GAPDH was used to verify equal protein loading. **C** and **D**, the two different cell lines were assessed for transformation ability in soft agar assay after treatment with 40 μM BaP for 1, 9 and 15 weeks. Cells (3×10^3^) were seeded in 1 ml of 0.3% Basal Medium Eagle (BME) agar containing 10% calf serum (CS). The cultures were maintained at 37°C in 5% CO_2_ atmosphere for 3 weeks and then colonies were counted using Image-Pro PLUS (v.6) software and representative images were shown **(C)**. The average colony number was calculated from three separate experiments and data were shown as mean±S.D. **(D)**. Significant differences were evaluated using the Student’s *t* test, and data were presented as mean±S.D. of triplicate experiments. The respective asterisks indicated a significant change in BaP-treated cells compared with the untreated controls (*, *p*< 0.05; **, *p*< 0.01) and # indicated a significant change between two different cells under the same condition (*p*<0.05).

To confirm the effective silencing of PARG in the shPARG cells, we examined the level of PARG expression in the shPARG and 16HBE cells which cultured at the 15th week by western blot. The expression of PARG was suppressed by 90% in the shPARG cells compared to the 16HBE cells (Fig 1A). To test whether BaP stimulates PAR protein synthesis, we analyzed PAR expression in shPARG cells at the 15th week after treated with 40 μM BaP for 24 hrs. We found that the level of PAR protein was greatly elevated in shPARG cells after BaP exposure (Fig 1B). To determine the role of PARG in cell transformation, we tested the colony formation of 16HBE and shPARG cells after treating with 40 μM BaP for 1, 9, and 15 weeks. We found that BaP exposure significantly increased colony formation in a time-dependent manner in both 16HBE and shPARG cells. However, the numbers of colony formed by shPARG cells were significantly less than that by 16HBE cells. Almost no colonies were observed in the control (Fig 1C and 1D, S1 Table). Overall, these data demonstrated that PARG played a key role in cell transformation induced by BaP.

### PARG silencing protected cells from BaP-induced DNA damage

**Fig 2:**
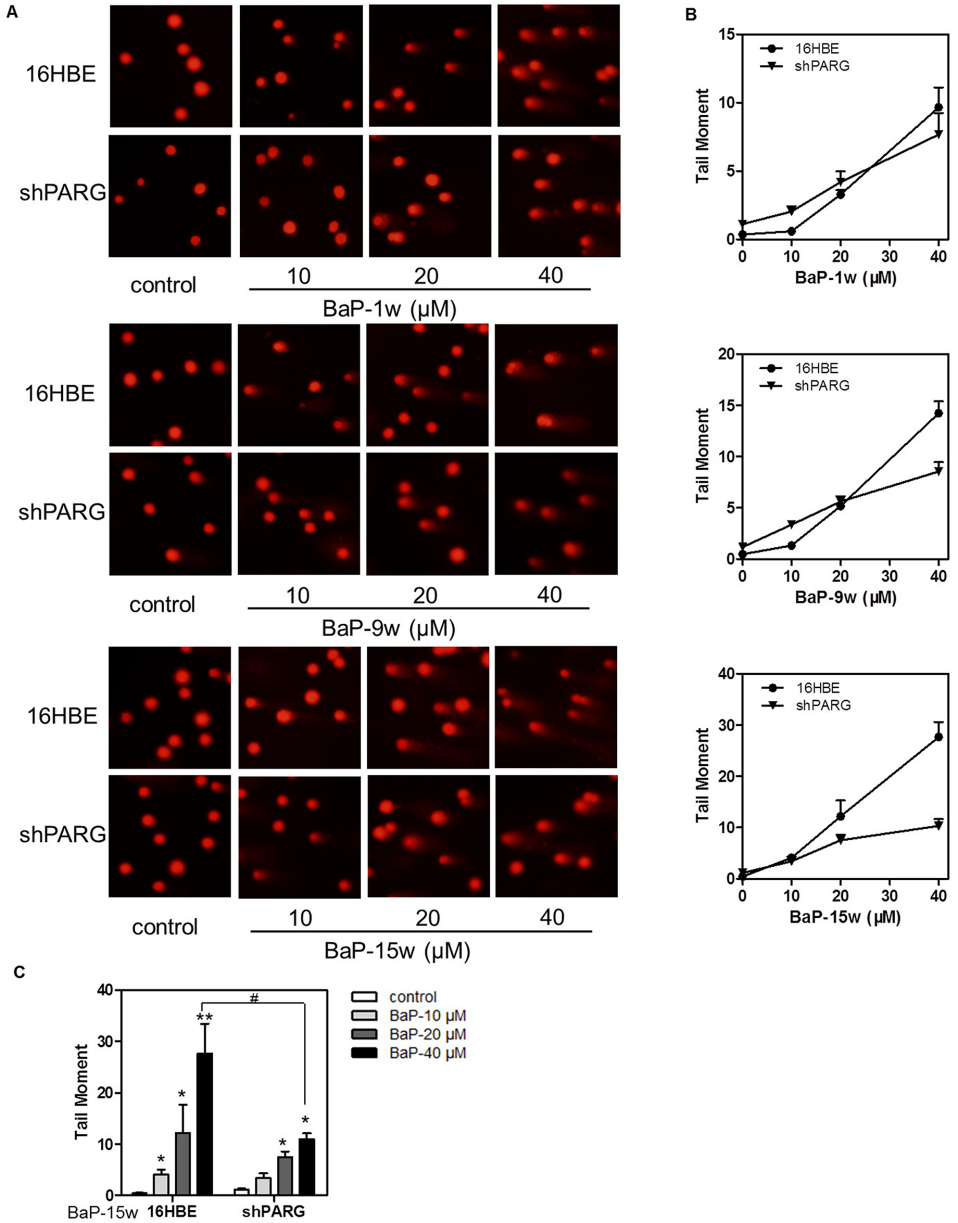
PARG silencing protected against BaP-induced DNA damage. DNA damage of cells was detected by comet assay after treatment with different concentrations of BaP for 1, 9 or 15weeks. Representative DNA comet images were shown **(A)** and the average tail moment was calculated from three separate experiments. Data were shown as mean±S.D. (**B** and **C**). Statistical differences were evaluated using the Student’s *t* test. The asterisks (* and **) indicated a significant difference (*p< 0.05 and **p<0.01, respectively) between the BaP-treated groups compared with untreated groups. ^#^ indicated a significant difference (*p*<0.05) between two different cells under the same condition.

To determine whether PARG silencing affects DNA damage during BaP-induced cell transformation, we performed the comet assay. We found that there was a concentration-dependent increase in DNA damage in both cell lines after BaP treatment (Fig 2A and 2B). The shPARG cells were less susceptible to BaP compared to the 16HBE cells, particularly at higher concentrations. The longest tails of comets were observed in the 16HBE cell groups while the tails of comets in the shPARG cells were relatively shorter. In addition, the 16HBE cells exposed to 40μM BaP for 15 weeks significantly (*p*<0.05) increased the tail moment of damaged cells as compared to the shPARG cells (Fig 2C, S2 Table). These results demonstrated that PARG silencing could partially prevent BaP induced DNA damage.

### PARG silencing reduced BaP-induced genomic instability

**Fig 3:**
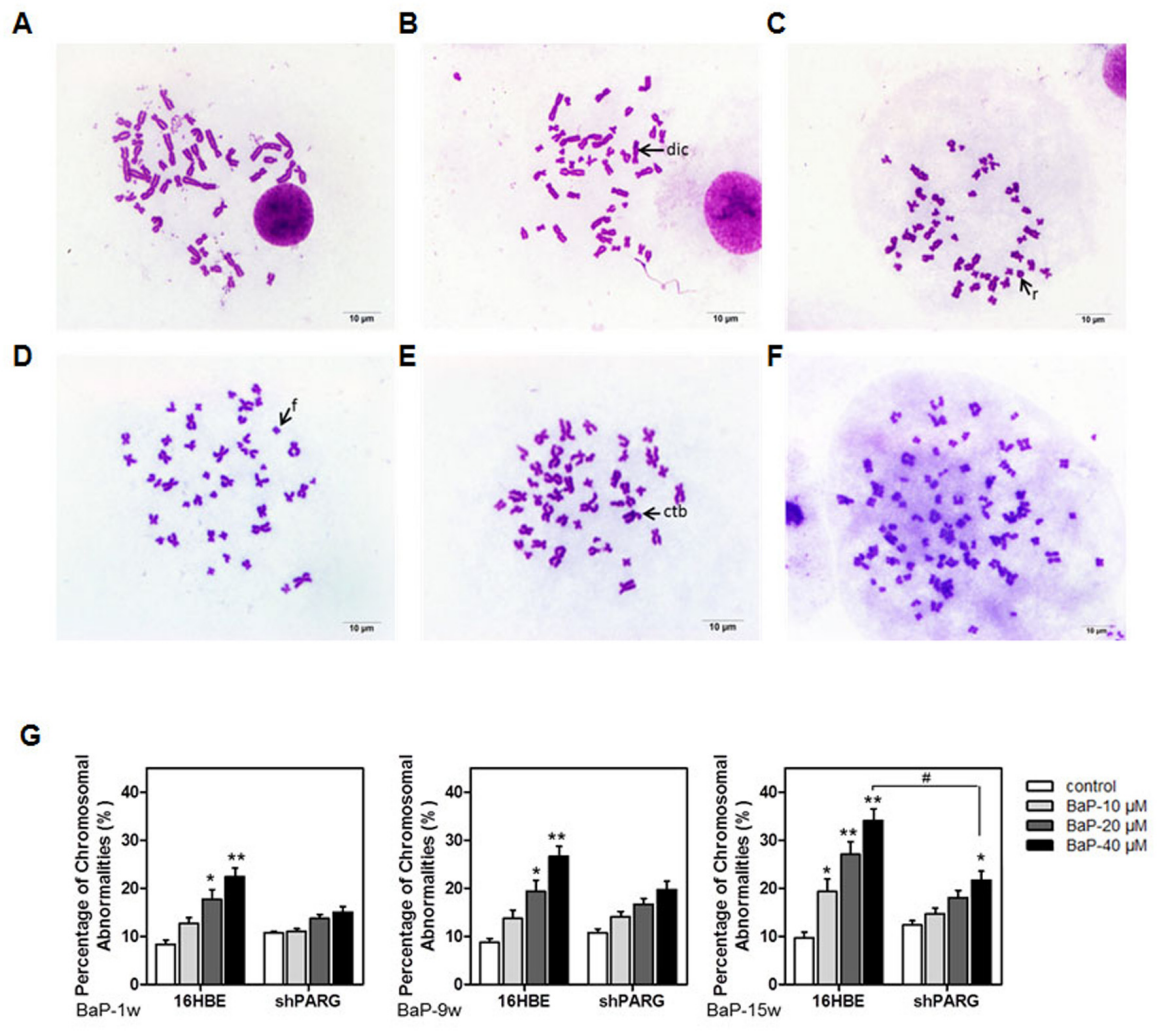
PARG silencing decreased BaP-induced chromosomal aberrations. Representative metaphase spread of cells stained with Wright-Giemsa stain. **A**, Normal chromosome; **B**, Dicentrics; **C**, Ring chromosome; **D**, Breaking of chromatid; **E**, Fragmentation; **F**, Polyploidy in chromosomes. **G**, The average of chromosomal aberrations was calculated from three separate experiments and data were shown as mean±S.D.. Statistical differences were evaluated using the Student’s *t* test and the asterisks (* and **) indicated a significant difference (* *p*<0.05 and ** *p*<0.01, respectively) between the BaP-treated groups compared with untreated groups. ^#^ indicated a significant difference (*p*<0.05) between two different cells under the same condition.

Unrepaired DNA damage leads to chromosome breakage which can promote genomic instability and contribute to carcinogenesis [26]. To examine whether silencing PARG alters the chromosome stability during BaP-induced cell transformation, we performed the chromosome aberration (CA) assay with Wright-Giemsa stain to assess the extent of DNA damage in both cell lines. A concentration- and time-dependent increase in the percentage of chromosomal damage was observed in both cell lines (Fig 3G, S3 Table). However, shPARG cells showed less chromosomal damage than 16HBE cells did. There was a slight increase of chromosomal aberration in shPARG cells after BaP treatment for 1 and 9 weeks, but it was not statistically significant (*p*>0.05). A significantly higher rate of chromosomal aberration was found in shPARG cells being treated with 40 μM BaP for 15 weeks (*p*<0.05). CAs induced by BaP included chromatid break (Fig 3B), chromosome break (Fig 3C), dicentrics chromosome (Fig 3D), ring chromosome (Fig 3E), and polyploidy (Fig 3F). In summary, PARG silencing had a protective effect to genomic integrity by decreasing BaP-induced chromosome aberration.

**Fig 4:**
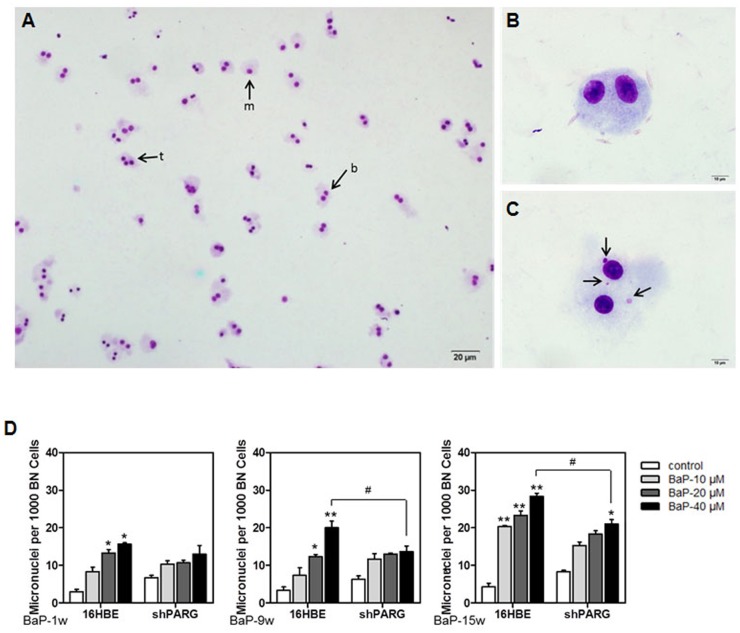
PARG silencing decreased BaP-induced micronuclei formation. Representative photo micrographs were shown as following: **A**, Normal mononucleated (m), binucleated (b), andtrinucleated (t) cells without micronuclei (100×). **B**, Binucleated cell without micronuclei (1000×). **C**, Multiple micronuclei (arrows) in binucleated cells (1000×). **D**, The average of micronuclei in binucleated cells was calculated from three separate experiments and data were shown as mean±S.D.. Statistical differences were evaluated using the Student’s *t* test and asterisks (* and **) indicated a significant difference (* *p*<0.05 and ** *p*<0.01, respectively) between the BaP-treated groups compared with untreated groups. ^#^ indicated a significant difference (*p*<0.05) between two different cells under the same condition.

The cytokinesis-blocked micronuclei assay (CBMN) is one of the most commonly used methods to measure DNA damage and may serve as an indicator for malignant transformation in cells (Fig 4). The total number of spontaneous micronuclei were increased in a concentration-dependent manner in both 16HBE and shPARG cell lines. The number of MN was significantly higher in 16HBE cells after treated by 40 μM BaP for 9 and 15 weeks as compared to that in shPARG cells (*p*<0.05) (Fig 4D, S4 Table). These results demonstrated that PARG silencing inhibited BaP-induced micronuclei formation, suggested PARG silencing protected cells against BaP-induced cytotoxicity and cytogenetic damage, and inhibited BaP-induced cell transformation by reducing genomic instability in cells.

### PARG silencing inhibited BaP-induced cell motility

**Fig 5:**
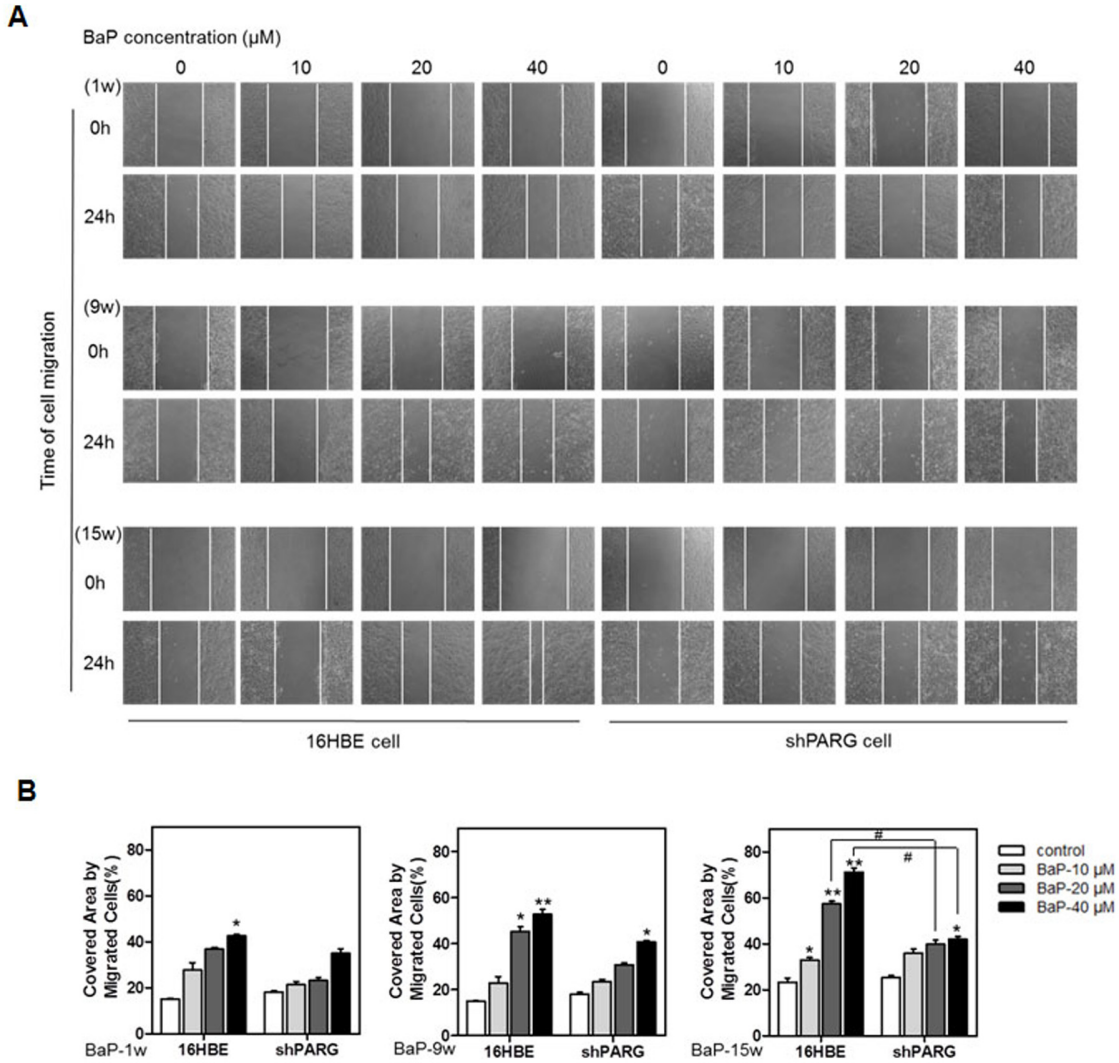
PARG silencing inhibited BaP-induced cell motility. Wound-healing assay of cells treated with different concentrations BaP for 1, 9 or 15 weeks. Representative images of cell migration were shown in **A**. Migration of the wound edge was measured at ten randomly chosen points in the photograph. The results were quantified as shown in **B**. Cell migration distances after 24 hrs ten randomly chosen points were compared with the distances at 0 h. Data were shown as mean±S.D. of triplicate measurements. *P* values were determined by Student’s *t* test. (* *p*<0.05 and ** *p*<0.01, BaP-treated groups compared with untreated groups; ^#^ *p*<0.05, a significant difference between two different cells under the same condition).

To investigate the role of PARG in cell motility induced by BaP, we performed the wound-healing assay. We found that cell migration was activated in both cell lines after BaP treatment. However, shPARG cells were migrated in a significantly slower rate than 16HBE cells (Fig 5, S5 Table). Wound closures were (42.60±1.22)%, (52.65±3.89)%, and (71.13±3.78) % in the 16HBE cell groups (40 μM BaP treatment for 1, 9, and 15 weeks of BaP), while wound closures were (34.90±2.04)%, (40.60±0.66)%, (41.93±1.29)% in the shPARG cell groups. In our previous study [21], we found that there was no significant difference in the proliferation rate between the two cell lines. However, BaP caused a concentration-dependent increase in cell death. These results indicated that PARG silencing significantly inhibited the migration of 16HBE cells during the process of BaP-induced cell transformation.

### PARG silencing suppressed BaP induced tumor formation in nude mice

**Fig 6:**
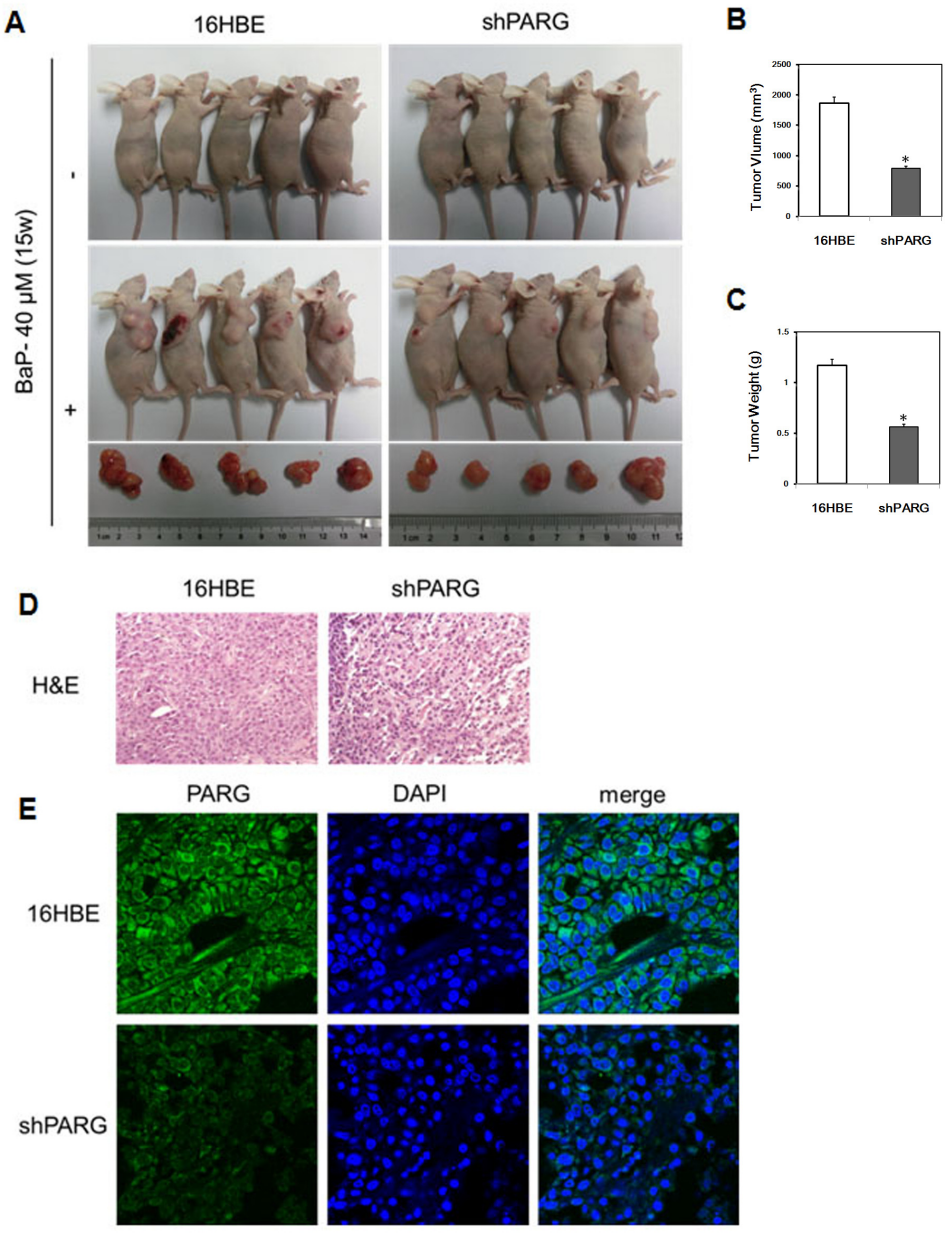
PARG silencing suppressed tumor formation of BaP-induced transformation cells in nude mice. Cells transformed by BaP exposure had tumorigenic activity in nude mice. The 16HBE cells and shPARG cells induced by 40 μM BaP for 15 weeks and untreated control cells were injected subcutaneously in nude mice. The nude mice were photographed after cells injection for 4 weeks and the tumors were excised **(A)**. Average tumor volume **(B)** and weight **(C)** were shown. Data were represented as mean±S.D. and significant differences were evaluated using the Student’s *t* test (* *p* < 0.05, BaP-treated groups compared with untreated groups; ^#^ *p*<0.05, a significant difference between two different cells under the same condition). **D**, H&E stained of the tumor tissue and imaged by the Image-Pro PLUS (v.6) computer software program (400×). **E**, Protein expression of PARG (green) in tumors from nude mice was examined by immunofluorescence staining and DNA was stained with DAPI (blue). Scale bar:20 μm.

To determine whether PARG silencing affects BaP induced tumor formation *in vivo*, we implanted BaP induced tumor cells into Balb/c nude mice (n = 5 mice per group). Tumors were developed from both BaP-induced transformed cell groups while no visible tumor in two control cell groups (p<0.05). Notably, the tumors formed by BaP-induced transformed shPARG cells were significantly smaller than that by BaP-induced transformed 16HBE cells (p<0.05) (Fig 6A and 6C, S6 Table). In addition, the rate of tumor growth was significantly slower in mice injected with BaP-induce transformed shPARG cells than 16HBE cells (p<0.05) (Fig 6B). Hematoxylin-eosin (H&E) staining revealed that the tumor developed from BaP-induce transformed shPARG cells had a lower cellularity and a higher rate of apoptosis than the tumor from 16HBE cells (Fig 6D).

To confirm whether PARG expression in tumors developed from BaP-induced transformed shPARG cells is still suppressed, we assessed the expression of PARG protein in tumors from nude mice using immunofluorescence analysis. We found that the tumors formed by shPARG cells showed a dramatically lower level of PARG protein expression than the tumor formed by 16HBE cells (Fig 6D). Taken together, these results provided strong evidences to show that PARG silencing suppressed tumor development from BaP-induced transformed cells *in vivo*.

## Discussion

BaP is known as a strong mutagen in both *in vitro* and *in vivo* assays, as well as a human carcinogen [20]. Chronic exposure to BaP can induce malignant transformation of human bronchial epithelial (16HBE) cells [27]. Chemical carcinogen induced transformation *in vitro* is highly correlated to carcinogenesis *in vivo*. Hence, it is a valuable method for assessing the carcinogenic potential of environmental chemicals [28]. To better understand the role of PARG during BaP carcinogenesis, we exposed human bronchial epithelial cells to BaP to cause malignant transformation of cells and then examined the toxic responses of PARG-deficient and the normal cells. In this study, we examined the expression of PARG in shPARG cells and demonstrated efficient suppression of PARG after 15 weeks of cell culture, which provided an important basis for subsequent research. The investigation of PAR formation by western blot revealed that BaP could stimulate PAR synthesis and confirmed that PARG was a major effector in response to BaP, which allowed us to highlight the role of PARG in the cellular response to long-term BaP stimulation.

DNA damage by chemicals is thought to constitute an essential molecular mechanism in the initiation of cancer [29]. In this study, our results identified that exposure to BaP significantly increased DNA damage of cells in a dose- and time-dependent manner, while PARG silencing protected cells against DNA damage caused by long-term BaP exposure. Studies showed that PARP catalyzes the polyADP-ribosylation (PARylation) of proteins involved in DNA damage repair [30]. Using PARG inhibitor to suppress PARG activity facilitated oxidative damage-induced PARylation as well as DNA damage repair [31]. PARG silencing kept the PAR level at a relatively high level under genotoxic stress, which facilitated DNA repair [32]. Our data confirmed that silencing of PARG in 16HBE cells lead to an accumulation of PAR under BaP stress and facilitated DNA damage repair.

Structural chromosomal aberrations (CAs) arise as consequences of direct DNA damage or replication errors from a damaged DNA template [33]. Micronuclei (MN) originate from chromosome breaks or chromosomes that fail to engage with the mitotic spindle when the cell divides [34]. DNA replication stress can cause genetic mutations, rearrangements, and chromosome mis-segregation, which lead to genomic instability and contribute to the development of cancer. Our data here showed that BaP led to a increase of CA and MN in 16HBE cells. This was in agreement with a previous study that demonstrated BaP induced carcinogenic transformation of normal cells by inducing genetic mutations and CA [35]. Recent efforts have been focused on developing chemical inhibitors of PARG to modulate poly(ADP-ribose) homeostasis in order to develop therapeutic strategies to treat various pathological conditions [36,37]. Homeostasis of PARlyation is important for an efficient repair of damaged DNA replication forks, thereby stabilizing the genome and preventing carcinogenesis [38]. In this study, we found that PARG silencing led to a decrease of CA and MN in cells treated by long-term BaP stimulation. This suggested that PARG silencing played an important role in the maintenance of genomic stability and prevented BaP carcinogenesis.

In this report, our results showed that PARG silencing significantly inhibited colony formation and cell migration induced by BaP, and suppressed tumor formation caused by BaP-induced cell transformation in nude mice. Recent findings suggest PARG silencing can inhibit the ability of cell migration, proliferation, and invasion [17,32]. Our previous studies have shown that PARG silencing down-regulated TCTP and Cofilin-1, which are genes associated with metastasis [39]. Our data in this study further elucidated the role of PARG in carcinogenesis induced by BaP. The results provided a novel insight into the possibility of targeting PARG in BaP induced cancer.

## Conclusions

In summary, our study provided novel evidences to support that PARG played an important role in BaP-induced cell transformation. PARG silencing significantly inhibited DNA damage, chromosome aberration, cell migration, and colony formation by decreasing the degradation of PAR, which inhibited tumorigenesis. This study suggested that PARG could be an attractive interventional target for BaP induced cancer.

## Supporting Information

S1 Table Numbers of colony in soft agar assay (means±S.D.,n = 5). (DOC)

S2 Table Average tail moment of DNA comets by comet assay (means±S.D.,n = 3). (DOC)

S3 Table Percentage of chromosomal abnormalities in different groups (%, means±S.D.,n = 3). (DOC)

S4 Table Number of micronuclei in different groups (means±S.D.,n = 3). (DOC)

S5 Table Covered area of migrated cells from different groups (%, means±S.D.,n = 3). (DOC)

S6 Table Tumor volume and weight of different groups (means±S.D.,n = 5). (DOC)
