# Supplementary material for: Poly(ADP-Ribose) Glycohydrolase (PARG) Silencing Suppresses Benzo(a)pyrene Induced Cell Transformation
Source: PLoS One. 2016 Mar 22;11(3):e0151172. doi: 10.1371/journal.pone.0151172 (PMC4803271; doi:10.1371/journal.pone.0151172)
Supplement: S1 Table — (DOC) [file pone.0151172.s001.doc]

**S1 Table. Numbers of colony in soft agar assay (means±S.D., n=5).**

| **Group** | **16HBE** | **shPARG** |
| --- | --- | --- |
| **untreated** | 95.80±4.35 | 85.20±3.54 |
| **1 W** | 105.80±4.27 | 87.80±3.02 |
| **9 W** | 291.80±27.46^b^ | 193.40±19.66^a^ |
| **15 W** | 703.40±65.45^b^ | 417.40±43.74^b,c^ |

Colony formation of two different cells induced by 40 μM BaP for 1, 9 or 15 weeks was detected by soft agar assay.

^a^ indicated a significant change (*p*<0.05) in BaP-treated cells compared with the untreated control.

^b^ indicated a significant change (*p*<0.01) in BaP-treated cells compared with the untreated control.

^c^ indicated a significant change (*p*<0.05) between two different cells under the same condition.
