# Supplementary material for: Poly(ADP-Ribose) Glycohydrolase (PARG) Silencing Suppresses Benzo(a)pyrene Induced Cell Transformation
Source: PLoS One. 2016 Mar 22;11(3):e0151172. doi: 10.1371/journal.pone.0151172 (PMC4803271; doi:10.1371/journal.pone.0151172)
Supplement: S2 Table — (DOC) [file pone.0151172.s002.doc]

**S2 Table.Average tail moment of DNA cometsby comet assay (means±S.D.,n=3).**

| **BaP(μM)** | **16HBE** | **shPARG** |
| --- | --- | --- |
| **0** | 0.44±0.06 | 1.16±0.12 |
| **10** | 4.09±0.54^a^ | 3.40±0.91 |
| **20** | 12.19±3.05^a^ | 7.53±1.04^a^ |
| **40** | 27.65±2.94^b^ | 10.32±1.32^a,c^ |

DNA damage of two different cells was detected by comet assay after treatment with different concentrations BaP for 15weeks.

^a^ indicated a significant change(*p*<0.05) in BaP-treated cells compared with the untreated control.

^b^ indicated a significant change(*p*<0.01) in BaP-treated cells compared with the untreated control.

^c^ indicated a significant change(*p*<0.05)between two different cells under the same condition.
