# Supplementary material for: Poly(ADP-Ribose) Glycohydrolase (PARG) Silencing Suppresses Benzo(a)pyrene Induced Cell Transformation
Source: PLoS One. 2016 Mar 22;11(3):e0151172. doi: 10.1371/journal.pone.0151172 (PMC4803271; doi:10.1371/journal.pone.0151172)
Supplement: S3 Table — (DOC) [file pone.0151172.s003.doc]

**S3 Table. Percentage of chromosomal abnormalities in different groups (%, means±S.D.,n=3).**

| **BaP**  **(μM)** | **1 W** | | **9 W** | | **15 W** | |
| --- | --- | --- | --- | --- | --- | --- |
|  | **16HBE** | **shPARG** | **16HBE** | **shPARG** | **16HBE** | **shPARG** |
| **0** | 8.33±0.88 | 10.67±0.33 | 8.67±0.88 | 10.67±0.88 | 9.67±1.45 | 12.33±0.88 |
| **10** | 12.67±1.20 | 11.00±0.58 | 13.67±1.76 | 14.00±1.15 | 19.33±2.60^a^ | 14.67±1.20 |
| **20** | 17.67±2.03^a^ | 13.67±0.88 | 19.33±2.33^a^ | 16.67±1.20 | 27.00±2.65^b^ | 18.00±1.53 |
| **40** | 22.33±1.86^b^ | 15.00±1.15 | 26.67±2.03^b^ | 19.67±1.76 | 34.00±2.52^b^ | 21.67±1.86^a,c^ |

Chromosome aberrations (CAs) assay of two different cells treated with different concentrations BaP for 1, 9 or 15 weeks.

^a^ indicated a significant change (*p*<0.05) in BaP-treated cells compared with the untreated control.

^b^ indicated a significant change (*p*<0.01) in BaP-treated cells compared with the untreated control.

^c^ indicated a significant change (*p*<0.05) between two different cells under the same condition.
