# Supplementary material for: Poly(ADP-Ribose) Glycohydrolase (PARG) Silencing Suppresses Benzo(a)pyrene Induced Cell Transformation
Source: PLoS One. 2016 Mar 22;11(3):e0151172. doi: 10.1371/journal.pone.0151172 (PMC4803271; doi:10.1371/journal.pone.0151172)
Supplement: S4 Table — (DOC) [file pone.0151172.s004.doc]

**S4 Table. Number of micronuclei in different groups (means±S.D., n=3).**

| **BaP**  **(μM)** | **1 W** | | **9 W** | | **15 W** | |
| --- | --- | --- | --- | --- | --- | --- |
|  | **16HBE** | **shPARG** | **16HBE** | **shPARG** | **16HBE** | **shPARG** |
| **0** | 3.00±0.58 | 6.67±0.66 | 3.33±0.91 | 6.33±0.88 | 4.33±0.88 | 8.33±0.33 |
| **10** | 8.33±1.20 | 10.33±0.88 | 7.33±2.048 | 11.67±1.44 | 20.33±0.33^b^ | 15.33±0.88 |
| **20** | 13.33±0.88^a^ | 10.67±0.66 | 12.33±0.58^a^ | 13.00±0.33 | 23.33±1.20^b^ | 18.33±0.88 |
| **40** | 15.67±0.33^a^ | 13.00±2.31 | 20.00±1.78^b^ | 13.67±1.48^c^ | 28.33±0.88^b^ | 21.00±1.15^a,c^ |

Cytokinesis-block micronuclei (CBMN) assay of two different cells treated with different concentrations BaP for 1, 9 or 15 weeks.

^a^ indicated a significant change (*p*<0.05) in BaP-treated cells compared with the untreated control.

^b^ indicated a significant change (*p*<0.01) in BaP-treated cells compared with the untreated control.

^c^ indicated a significant change (*p*<0.05) between two different cells under the same condition.
