# Supplementary material for: Poly(ADP-Ribose) Glycohydrolase (PARG) Silencing Suppresses Benzo(a)pyrene Induced Cell Transformation
Source: PLoS One. 2016 Mar 22;11(3):e0151172. doi: 10.1371/journal.pone.0151172 (PMC4803271; doi:10.1371/journal.pone.0151172)
Supplement: S5 Table — (DOC) [file pone.0151172.s005.doc]

**S5 Table. Covered area of migrated cells from different groups (%, means±S.D., n=3).**

| **BaP**  **(μM)** | **1 W** | | **9 W** | | **15 W** | |
| --- | --- | --- | --- | --- | --- | --- |
|  | **16HBE** | **shPARG** | **16HBE** | **shPARG** | **16HBE** | **shPARG** |
| **0** | 15.01±0.32 | 17.99±0.64 | 15.04±0.14 | 18.12±0.80 | 23.34±1.77 | 25.36±0.86 |
| **10** | 27.61±3.30 | 21.29±1.43 | 22.92±2.74 | 23.38±1.13 | 32.81±1.22^a^ | 35.94±1.84 |
| **20** | 36.68±0.90 | 23.24±1.17 | 45.19±2.07^a^ | 30.69±0.92 | 57.36±1.19^b^ | 39.87±1.70^c^ |
| **40** | 42.60±0.71^a^ | 34.90±2.04 | 52.65±2.25^b^ | 40.60±0.66^a^ | 71.13±1.89^b^ | 41.93±1.29^a, c^ |

Wound-healing assay of two different cells treated with different concentrations BaP for 1, 9 or 15 weeks.

^a^ indicated a significant change (*p*<0.05) in BaP-treated cells compared with the untreated control.

^b^ indicated a significant change (*p*<0.01) in BaP-treated cells compared with the untreated control.

^c^ indicated a significant change (*p*<0.05) between two different cells under the same condition.
