# Supplementary material for: Poly(ADP-Ribose) Glycohydrolase (PARG) Silencing Suppresses Benzo(a)pyrene Induced Cell Transformation
Source: PLoS One. 2016 Mar 22;11(3):e0151172. doi: 10.1371/journal.pone.0151172 (PMC4803271; doi:10.1371/journal.pone.0151172)
Supplement: S6 Table — (DOC) [file pone.0151172.s006.doc]

**S6 Table. Tumor volume and weight of different groups (means±S.D., n=5).**

| **Group** | **Volume (mm^3^)** | **Weight (g)** |
| --- | --- | --- |
| **16HBE** | 1867.53±126.60 | 1.17±0.13 |
| **shPARG** | 786.86±69.10^a^ | 0.56±0.09^a^ |

Tumor volume and weight were measured after implantation for 4 weeks.

^a^ indicated a significant difference (p<0.05) between two different cells.
